# RACIAL/ETHNIC DIFFERENCES IN LONELINESS AMONG OLDER ADULTS: THE ROLE OF INCOME AND EDUCATION AS MEDIATORS

**DOI:** 10.1093/geroni/igae098.1891

**Published:** 2024-12-31

**Authors:** Harry Taylor, Arka Roy, Kazumi Tsuchiya, Ann Nguyen, Yu-Chih Chen, Thomas Cudjoe, Weidi Qin

**Affiliations:** University of Toronto, Toronto, Ontario, Canada; University of Toronto, Toronto, Ontario, Canada; University of Toronto, Toronto, Ontario, Canada; Case Western Reserve University, Cleveland, Ohio, United States; The University of Hong Kong, Hong Kong, China (People’s Republic); Johns Hopkins University School of Medicine, Baltimore, Maryland, United States; University of Wisconsin Madison, Madison, Wisconsin, United States

## Abstract

Our study examines differences in loneliness by race/ethnicity among older adults in the United States and whether income and education mediate these differences. Data came from the Health and Retirement Study Leave-Behind Questionnaire, 2014-2016. Loneliness was measured by the UCLA 3-item loneliness scale. Race/ethnicity categories were White, Black, and Hispanic/Latino. The mediator variables were total household income and education. Sociodemographic covariates included gender, age, employment status, living arrangements, country region, urbanicity, and HRS LBQ Wave. Multivariable linear regression models were used to determine differences in loneliness by race/ethnicity. The KHB mediation method was used to determine if income and education mediated racial/ethnic differences in loneliness. In bivariable analyses, White and Hispanic/Latinx older adults had comparable levels of loneliness, while Black older adults had higher loneliness scores. After multivariable adjustment, Hispanic/Latinx older adults had the lowest levels of loneliness while White and Black older adults had comparable levels of loneliness. A complete mediation was found between Whites and Blacks, in that income and education completely mediated differences in loneliness between these groups. A partial mediation was found between Whites and Hispanics, and between Blacks and Hispanics. Income as a mediator accounted for greater racial/ethnic differences in loneliness compared to education. Our study is the first to explicitly determine if socioeconomic factors mediate race/ethnicity differences in loneliness among a nationally representative sample of older adults. Study findings can inform evidence-based interventions and programs to reduce loneliness among older adults.

